# Increased Axonal Ribosome Numbers Is an Early Event in the Pathogenesis of Amyotrophic Lateral Sclerosis

**DOI:** 10.1371/journal.pone.0087255

**Published:** 2014-01-30

**Authors:** Mark H. G. Verheijen, Marco Peviani, Rita Hendricusdottir, Erin M. Bell, Martin Lammens, August B. Smit, Caterina Bendotti, Jan van Minnen

**Affiliations:** 1 Department of Molecular and Cellular Neurobiology, Center for Neurogenomics and Cognitive Research, Neuroscience Campus Amsterdam, VU University, Amsterdam, The Netherlands; 2 Laboratory of Molecular Neurobiology, Department of Neuroscience, “Mario Negri” Institute for Pharmacological Research, Milan, Italy; 3 Hotchkiss Brain Institute, University of Calgary, Calgary, Canada; 4 Department of Pathology, Antwerp University Hospital, University of Antwerp, Antwerp, Belgium; University of Edinburgh, United Kingdom

## Abstract

Myelinating glia cells support axon survival and functions through mechanisms independent of myelination, and their dysfunction leads to axonal degeneration in several diseases. In amyotrophic lateral sclerosis (ALS), spinal motor neurons undergo retrograde degeneration, and slowing of axonal transport is an early event that in ALS mutant mice occurs well before motor neuron degeneration. Interestingly, in familial forms of ALS, Schwann cells have been proposed to slow disease progression. We demonstrated previously that Schwann cells transfer polyribosomes to diseased and regenerating axons, a possible rescue mechanism for disease-induced reductions in axonal proteins. Here, we investigated whether elevated levels of axonal ribosomes are also found in ALS, by analysis of a superoxide dismutase 1 (SOD1)^G93A^ mouse model for human familial ALS and a patient suffering from sporadic ALS. In both cases, we found that the disorder was associated with an increase in the population of axonal ribosomes in myelinated axons. Importantly, in SOD1^G93A^ mice, the appearance of axonal ribosomes preceded the manifestation of behavioral symptoms, indicating that upregulation of axonal ribosomes occurs early in the pathogenesis of ALS. In line with our previous studies, electron microscopy analysis showed that Schwann cells might serve as a source of axonal ribosomes in the disease-compromised axons. The early appearance of axonal ribosomes indicates an involvement of Schwann cells early in ALS neuropathology, and may serve as an early marker for disease-affected axons, not only in ALS, but also for other central and peripheral neurodegenerative disorders.

## Introduction

Amyotrophic lateral sclerosis or Lou Gehrig’s disease is a devastating illness affecting motor neurons in the motor cortex, brain stem and spinal cord, leading to axon degeneration, muscle atrophy, paralysis and ultimately death of the patient. For most cases (90%) the origin of the disease is not known (sporadic [s]ALS), whereas mutations in the ubiquitously expressed Cu/Zn superoxide dismutase (SOD) 1 are at the basis of 20% of the familial cases of ALS [1]. A common denominator for both sALS and fALS is the presence of protein aggregates in neuronal somata and axons [2]. However, in spite of 130 years of research the mechanism underlying the selective motor neuron degeneration remains elusive [3].

The SOD1^G93A^ transgenic mouse, in which the mutation is ubiquitously expressed, is a widely used rodent model to study fALS [1]. In these animals many features of the human disease are observed such as motor neuron loss, aggregate formation and paralysis, and reduced axonal transport [4]. Progression of the disease follows a predictable pattern, and allows analysis of neurons and axons in defined stages of the disease [1], [5]. Studies using cell specific expression of mutant SOD1 showed that the detrimental effects of SOD1 are due to a newly acquired toxicity independent of its dismutase activity [6]. Mutated SOD1 expression in motor neurons, but also in neighboring microglia [7] and astrocytes [8] leads to disease progression. In contrast, accumulation of mutant SOD1 in Schwann cells does not lead to ALS symptoms in transgenic mice [9]. Together these data indicate a role for SOD1 in glial cells in disease progression, although the exact mode of action remains elusive.

In inherited polyneuropathies belonging to the Charcot Marie Tooth (CMT) class I, mutant gene expression in Schwann cells, especially of myelin genes, often leads to axonal degeneration [10] underlining the intimate interdependence between glia and axons. In relation to this, we recently showed for CMT classes I and II that peripheral axons of patients often show an abundance of polyribosomes, a condition not seen in patients that do not have a history of peripheral nerve disorders [11]. Although these neuropathies, including motor neuron diseases, are heterogeneous in the processes that underlie the pathophysiology, a common feature is that axons show impaired axonal transport [12], [13], [14] resulting in a diminished supply of proteins in downstream axonal areas. This condition is akin to axons that are not longer connected to their soma, in which we also observed an increase in axonal ribosomes that were supplied by the periaxonal Schwann cells [15].

To investigate whether axons affected by motor neuron diseases show similar phenomena of increased abundance of ribosomes, we investigated peripheral axons of a mouse model for the upper motor neuron disease ALS and of a patient suffering from sALS, for the presence of axonal ribosomes.

Our analyses of peripheral nerves from presymptomatic and symptomatic animals and the ALS patient show an increased number of ribosomes in axons, and the presence of polyribosome-containing myelin-membrane vesicles in axons indicative for a Schwann cell origin. These data demonstrate that polyribosomes enter the axonal compartment well before the onset of behavioral deficits, suggesting a role for Schwann cells early in ALS. The presence of axonal ribosomes may serve as an early biomarker in ALS, and potentially also in other neurodegenerative disorders.

## Materials and Methods

### Animals

Procedures involving animals and their care were in accordance to international laws and policies (EEC Council Directive 86/609, OJ L 358, 1 Dec.12, 1987; NIH Guide for the Care and use of Laboratory Animals, U.S. National Research Council, 1996). The Mario Negri Institute (Milan, Italy) approved the experiments, which were conducted according to the institutional guidelines, which are in compliance with Italian laws (D.L. no. 116, G.U. suppl. 40, Feb. 18, 1992, Circular No.8, G.U., 14 luglio 1994). At the VU University Amsterdam the experiments were approved by the institutional ethic committee of the VU University (Animal Experimentation Committee). All animals were housed and bred according to the institutional and Dutch governmental guidelines for animal welfare. At the University of Calgary permission was obtained by Health Science Animal Care Committee (HSACC), which adhered strictly to guidelines set by the Canadian Council on Animal Care.

Mice were maintained at a temperature of 21±1°C with a relative humidity 55±10% and a light-dark cycle 12 h/12 h. Food (standard pellets) and water were supplied *ad libitum*. Transgenic SOD1^G93A^ mice expressing about 20 copies of mutant human *SOD1* with a Gly93Ala substitution (B6SJL-TgNSOD-1-SOD1^G93A^-1Gur) and mice overexpressing the human wild-type *SOD1* gene (*S*OD1wt) were originally obtained from Jackson Laboratories and maintained on a C57BL/6 genetic background at Harlan Italy S.R.L., Bresso (MI), Italy. For each experimental group, 5 animals were sacrificed. The different stages of the pathology were defined on the basis of our previous histopathological analyses and observation of motor performances in this animal model [1]. For analysis of motor performances, the mice were tested for extension reflex and on rotarod-apparatus and on grid once a week, starting by the age of 7 weeks. Pre-symptomatic mice (around 12 weeks of age) showed a normal performance both in rotarod and in grid test, as compared to non-transgenic (Ntg) littermates, which are identical in genetic make-up to wild-type (wt) animals. Early-symptomatic mice (around 17 weeks of age) showed a marked reduction of extension reflex and decline of about 20% in the grid test [16]. In studies on fALS ^SOD1G93A^ mice, tissues from mice overexpressing human wild type SOD1 (SOD1wt) were used as negative controls in addition to the Ntg littermates (12–14). SOD1wt mice do not show pathological changes up to 20 weeks of age [17], [18]. Animals were sacrificed at 12 and 17 weeks after birth, to obtain animals before and after the manifestation of progressive muscular weakness. Sciatic and phrenic nerves were dissected and fixed for either electron microscopy or immunocytochemistry, see below. From the phrenic nerve the distal 2 cm of the nerve trunk before the nerve branches into the diaphragm was dissected.

### Electron Microscopy

Phrenic and sciatic nerves of SOD1^G93A^ transgenic mice and their Ntg littermates were fixed O/N in 4% paraformaldehyde in phosphate buffered saline (PBS), postfixed in 2% glutaraldhyde and 1% OsO_4_ in 0.1 M cacodylate buffer, pH 7.4 for 2 h on ice, dehydrated in a graded series of alcohol and embedded in Epon. For quantification of ribosomes, the number of polyribosomes was quantified in 100 randomly chosen axons in ultrathin (70 nm) sections. Size determination of ribosomes was performed on high magnification EM images, using Image Track, available at http://www.ucalgary.ca/styslab/imagetrak, courtesy of Dr. P. K. Stys. Nerves from patients were fixed according to the protocol used in the Nijmegen institute from which the human material was obtained [19].

### Immunocytochemistry

Mice were sacrificed, in accordance with ethical procedures (see above), by decapitation. The sciatic and phrenic nerves of 12 and 17 week old SOD1^G93A^ mice, their littermates and SOD1wt mice (corresponding to the advanced stage of disease progression) were rapidly dissected and fixed O/N in 4% paraformaldehyde. After a brief rinse in PBS), the nerves were cryoprotected with 30% sucrose in for 24 h, embedded in OCT medium and frozen at −80°C. 14 µm cryostat sections were mounted on Superfrost™ slides. To detect ribosomes, slides were incubated O/N with a 1∶500 diluted anti-ribosomal P-protein antiserum (human anti-ribosome, Immunovision) or a mouse monoclonal anti-ribosomal RNA (Y10B, [20]). To outline the axons, slides were either incubated in the lipophilic red fluorescent dye Nile red (InVitrogen) or incubated with rabbit anti-myelin basic protein (Stem Cell Technologies, 1∶250 dilution). After several rinses in PBS the slides were incubated in the appropriate secondary Alexa-conjugated fluorescent antisera (Molecular Probes) and analyzed with a Zeiss 510 confocal microscope.

### In situ Hybridization

Nonradioactive in situ hybridization was carried out to detect the presence of rRNA in isolated axons using a 28S ribosomal RNA probe [21]. The method is described in detail in [22], however here we used an Alexa 488 conjugated goat-anti-digoxigenin (Invitrogen) antiserum to allow fluorescent detection of the hybridization signal. After in situ hybridization, myelin was labeled with the red fluorescent dye Nile red (diluted 1∶2500 in PBS for 30 min). Sections were imaged on a Nikon EZ-C1 confocal microscope.

### sALS Patient Data

Analyses were performed on a sural nerve autopsy of a 64-year-old man, diagnosed with sporadic ALS and who died of respiratory insufficiency. Electron microscope analysis of the sural nerve showed signs of axonal loss, but no active axonal degeneration. To quantify axonal ribosomes, 250 axons were analyzed for the presence of ribosomes. Written consent was obtained to use the patient’s tissue for scientific purposes.

### Statistics

The increase in ribosomes in phrenic nerve axons of SOD1^G93A^ transgenic animals was tested for statistical significance (n = 5 for each experimental group) using a one way analysis of variance followed by a multiple comparison post test (Tukey-Kramer).

## Results

### Axonal Ribosomes in ALS Mice

**Figure 1:**
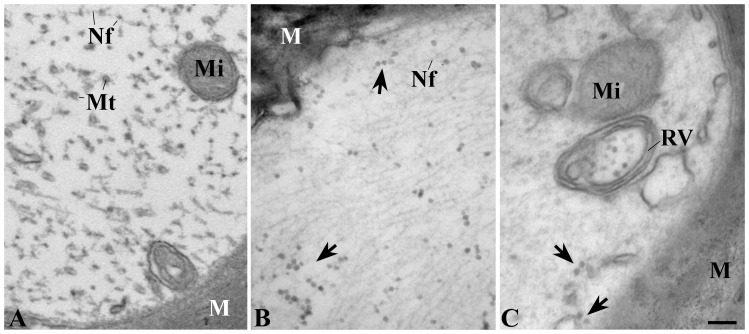
Increased axonal ribosome numbers in a mouse model for ALS. A, sciatic nerve from a control (non-transgenic) mouse showing mitochondria (Mi), cross-sectioned microtubules (Mt) and neurofilament fibers (Nf) in the axoplasm. Nf is longitudinally and cross-sectioned in this image. At first glance, cross-sectioned Nf might be confused with axonal ribosomes (see figure B), however Nf is considerably smaller and do not show the characteristic bead-like pattern of polyribosomes. B, SOD1^G93A^ transgenic mouse. Many polyribosomal clusters are present (arrows), showing a characteristic bead-like patterns (arrows). C, ribosomes are present in a vesicle with multiple membranes (RV, ribosome containing vesicle). Arrows point at ribosomes in axoplasm. M, myelin; Bars A–C, 100 nm.

**Figure 2:**
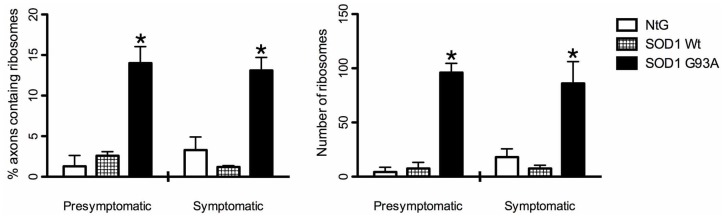
Quantification of increase of polyribosomes in myelinated axons in the phrenic nerve of SOD1^G93A^ mutant mice. Both the percentage of myelinated axons containing ribosomes (A) as well as the absolute number of ribosomes in axons (B) has increased dramatically in both presymptomatic (12 weeks PN, n = 5, *p<0.01) as well as symptomatic (17 weeks PN n = 5, *p<0.01) compared to their littermate controls (Ntg, n = 5) and SOD1 Wt animals, (n = 5) whereas there are no statistical differences between the presymptomatic and symptomatic groups.

We have previously shown that the number of ribosomes in axons of peripheral nerves increases in CMT diseases [11]. To determine whether a similar phenomenon occurs in (mouse models of) the upper motor neuron disease ALS, we investigated sciatic and phrenic nerves of symptomatic *SOD1^G93A^* mice for the presence of polyribosome-like structures. In contrast to controls (Figure 1A), abundant polyribosome-like structures were observed in the axonal compartment of myelinated axons in both the sciatic (Figure 1B) nerve and the phrenic nerve (Figure S1C). Furthermore, membrane-bound structures containing polyribosome-like particles were detected in the axoplasm of both nerves (Figure 1C, Figure S1D), similar to vesicles that we recently reported for desomatized axons [15]. In the axons of control Ntg littermates of the SOD1^G93A^ mice, and SOD1wt transgenic mice, polyribosomes were scarce, whereas polyribosome-containing vesicles were not detected (Figure 1A, Figure S1A, B). Quantitative analysis of axonal polyribosomes in myelinated phrenic nerve axons of pre-symptomatic (12 week old animals) and symptomatic SOD1^G93A^ mice (17 week old animals) showed a significant increase in SOD1^G93A^ mice (n = 5, *p<0.01), compared to Ntg controls and SOD1wt, in the number of myelinated axons that contain polyribosomes (Figure 2A) as well as in the total number of ribosomes in axons (Figure 2B). Importantly, this was not only observed for symptomatic mice, but also for pre-symptomatic animals. Furthermore, this increase was specific for myelinated axons: polyribosomes were virtually undetectable in unmyelinated axons of Ntg, SOD1wt and SOD1^G93A^ transgenic mice (data not shown).

**Figure 3:**
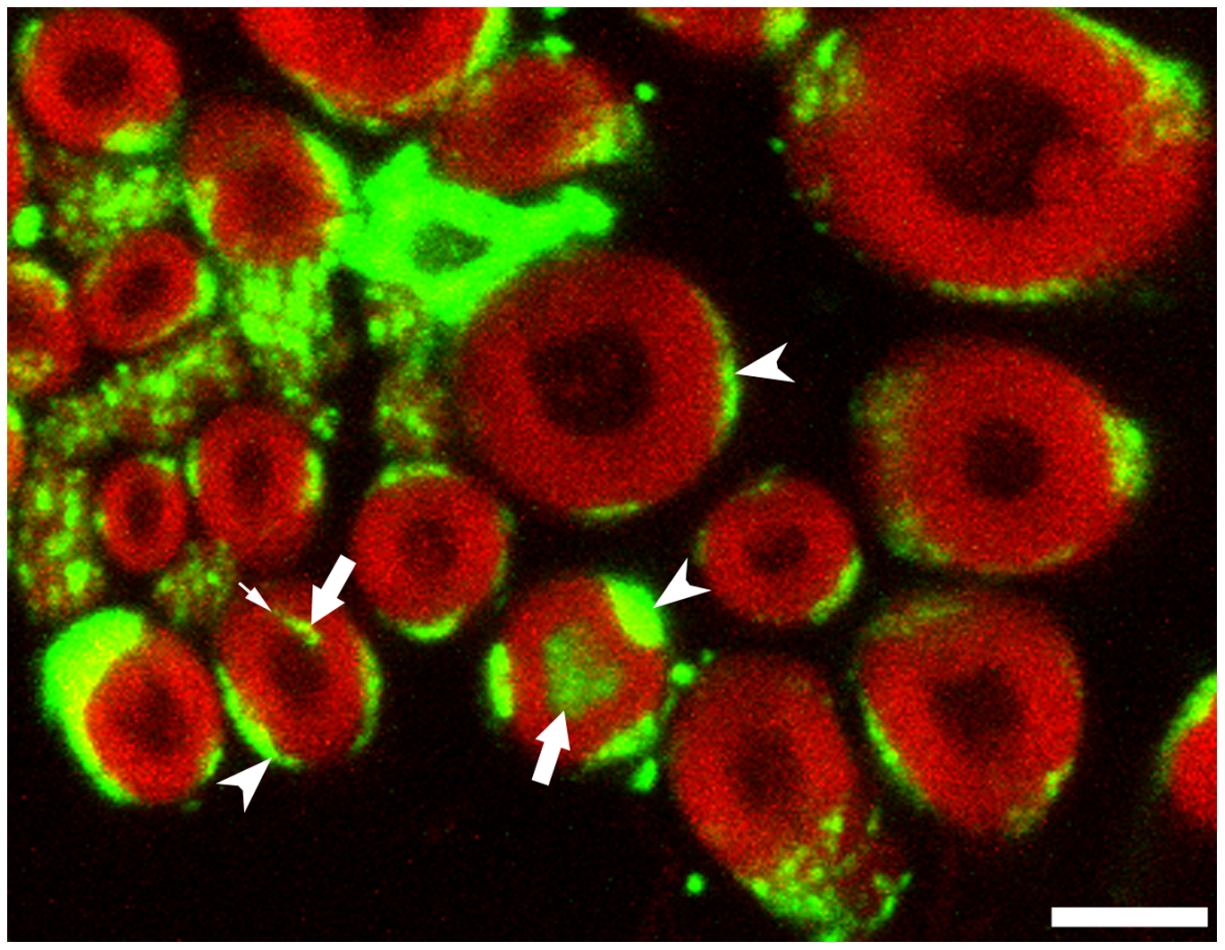
Symptomatic SOD1^G93A^ mice, sciatic nerve. Non-radioactive in situ hybridization for rRNA (green) yields strong signals in 2 myelinated axons (arrows), delineated by Nile red fluorescence of myelin (red). In the left axon, the small signal inside the myelin sheath (arrowhead) most likely corresponds to ribosomal RNA localized in ad-axonal Schwann cell cytoplasm or in Schwann cell cytoplasm between the myelin membranes (e.g. clefts of Schmidt-Lantermann), which has previously been suggested as a transfer route for ribosomes from Schwann cells to axons (Court et al, J Neurosci 2008, 28∶11024–11029). The signal outside the myelin sheets (arrowheads) corresponds to ribosomes in the peri-axonal Schwann cell cytoplasm. Please note that most axons do not show a hybridization signal, but show a strong Schwann cell signal (arrowheads), confirming the specificity of the ISH. See Figure S2 for optical sections of this area. Bar, 5 µm.

**Figure 4:**
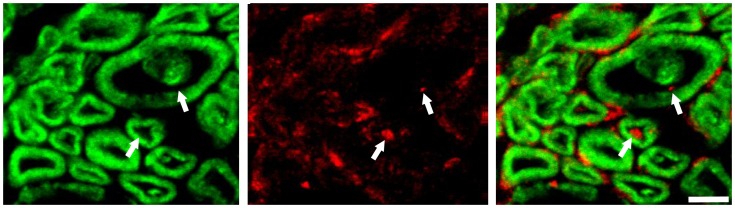
Symptomatic SOD1^G93A^ mice, phrenic nerve. Anti-ribosomal P-protein immunofluorescence (red) is present in myelinated axons outlined by anti-myelin basic protein antibody (arrows). Bar, 5 µm.

To confirm that the observed axonal particles in symptomatic SOD1^G93A^ mice are indeed ribosomes, we first applied *in situ* hybridization on ribosomal RNA and clearly detected rRNA in the sciatic nerve axoplasm (Figure 3, and Figure S2, 3). Second, using immunocytochemistry we found localization of ribosomal P-protein in the axoplasm of the phrenic nerve (Figure 4). Third, using a monoclonal antibody to ribosomal RNA (Y10B), we found a positive signal in myelinated axons of the sciatic nerve (Figure S4). The presence of axonal rRNA or ribosomal P-protein was only rarely observed for Ntg littermates and SOD1wt mice (data not shown). Together, both methods clearly identified ribosome-positive signals in axons of phrenic and sciatic nerves of SOD1^G93A^ mice at symptomatic as well as pre-symptomatic stage of the disease. Interestingly, in some cases axonal rRNA was found to co-localize with myelin immunofluorescence, both in the myelin sheath (Figure 3) as well as in the axon (Figure S5), suggesting that the ribosomes were localized in myelin containing structures, which is the likely vehicle to transfer polyribosomes to axons as suggested in [15].

Taken together, based on the immunocytochemical, in situ hybridization and quantitative EM analysis of SOD1^G93A^ mice, we conclude that ALS is associated with a significant increase in the number of ribosomes in myelinated axons. Importantly, we demonstrated that these axonal ribosomes appear early during ALS pathogenesis.

### Axonal Ribosomes in an ALS Patient

**Figure 5:**
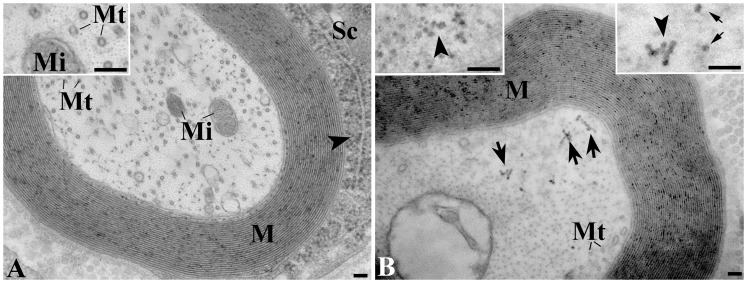
Increased axonal ribosome numbers in human ALS. A, sural nerve biopsy of a control person having no neurological symptoms, showing a myelinated axon in which no polyribosomes can be discerned. Polyribosomes are detected in the Schwann cell (Sc), either freely in the cytoplasm, or associated with the rough endoplasmic reticulum (arrowhead). Inset shows detail of axoplasm containing many cross sectioned microtubules (Mt). B, sural nerve autopsy from a patient diagnosed with sporadic ALS. In the axoplasm, clusters of polyribosomes are presented. Left inset: arrowhead points to ribosomes in the cytoplasm of a Schwann cell, which show similar morphology as those in the axoplasm (right inset). Bar, 100 nm.

Next, we determined whether in human ALS a similar phenomenon is present. We analyzed a sural nerve autopsy of a patient that was diagnosed with sALS based on loss of nerve function and evidence of axonal loss which is in line with the large majority of ALS patients which were shown to have a sensory nerve pathology after sural nerve biopsy [23]. The current EM analysis demonstrated polyribosome-like particles in axons of this nerve (Figure 5B), which were scarcely present in controls (Figure 5A). To strengthen the correct identification of these particles as axonal ribosomes, we measured the diameter of ribosome-like particles on EM images and compared their size with ribosomes in Schwann cells. The size of the ribosome-like particles in axons (19.2±2.0 nm, mean ± standard error) was found to be similar to those in the Schwann cell (19.8±2.0 nm), and to correspond to the published size of ribosomes of about 20 nm [11], [24]. Quantification showed that 11.0% of myelinated axons in this patient contained polyribosome-like particles. In contrast, axons from control patients (Figure 1B), i.e. persons that had no history of a nerve disorder, contained a considerably lower number of polyribosome-like particles (1.4±0.1%, n = 3).

## Discussion

The here presented data on a transgenic ALS mouse model and an ALS patient demonstrate a strong upregulation of polyribosomes in the axonal compartment of myelinated axons. In addition, in SOD1^G93A^ transgenic mice, a clear cut increase in the number of axonal ribosomes is detected both in pre-symptomatic as well as symptomatic mice, whereas the numbers in controls and SOD1wt were not increased. An important question concerns the origin of the ribosomes. According to the generally held opinion, ribosomes are transported to peripheral axonal domains by microtubule-based, fast axonal transport, often associated in ribonucleoprotein (RNP) particles [25], [26]. This mechanism might also account for the increase of axonal ribosomes in ALS. However, a common consequence of neuropathies is disruption of the ability of neurons to transport cargo along the entire length of the axon [12], [13], which includes CMTs and ALS [27], [28], [29]. This reduced axonal transport is also likely to affect the anterograde transport of ribosomes. An alternative source for axonal ribosomes might be the periaxonal Schwann cells as was recently demonstrated for injured and regenerating axons [15], [30]. These cells bud off so-called ribosome-containing vesicles from the peri-axonal Schwann cell cytoplasm into the axonal compartment [15]. In the current study we observed the presence of ribosome containing vesicles in axons from presymptomatic and symptomatic SOD1^G93A^ mice, which suggests that Schwann cells may be involved in the observed increase in axonal ribosomes. Glia cell to axon transfer of ribosomes was recently reported also to occur in intact nerves of rodents [25], [31], [32], [33]. Interestingly, Li and collaborators, in their ultrastructural study on spinal motor axons, showed similar ribosome-loaded vesicles in axons as in the present study, and the authors proposed that these vesicles are instrumental in transport of ribosomes from oligodendrocytes to axons [32].

Furthermore, our observation that ribosomal RNA was found colocalized within myelin, and with myelin-membrane vesicles in the axoplasm, supports the notion that ribosomes may be transported from Schwann cells to axons by vesicles containing myelin membranes, as we previously suggested [15]. These vesicles are dissimilar from exosomes that have been shown to be released from oligodendrocytes and Schwann cells. (For review see: [34] and [35], [36], [37]) in that they are considerably larger, and surrounded by multiple (myelin) membranes. Nevertheless, they may serve similar functions, i.e. providing trophic support for neurons. Recently, Schwann cell exosomes were shown to stimulate axon regeneration [37]. Although these studies indicate a glial to axon transport of molecules and organelles, they do not exclude the possibility that the parent cell body of the axon is (partly) responsible for these processes.

What could be the functional relevance of the increase in axonal ribosomes in disorders that damage axons? Polyribosomes consist of several ribosomes simultaneously translating a strand of mRNA and are considered the morphological correlate of ongoing protein synthesis [38]. This indicates that proteins are being synthesized in polyribosome-containing axons, however the identity of these proteins remains to be identified. That [36], [37]axons have the ability to synthesize a large variety of proteins, ranging from cytosolic to integral membrane proteins has been shown by several groups in the last decade [39], [40], [41], [42], [43], [44]. As already noted above, axonal degeneration is particularly observed in distal regions, suggested to be associated with decreased axonal transport [12], and because of the longer nerves in humans thought to occur to a larger extent in humans than in mice [14]. In demyelinating neuropathies, such as CMT1A, the lack of myelin sheath has pronounced effects on axonal transport [12], [27]. In axonal neuropathies many mutated genes are directly involved in axonal transport [45]. Furthermore, slowing of axonal transport is an early event observed in ALS-linked SOD1 mutant motor neurons, and occurs well before motor neuron degeneration is detected [29], [46]. In the above mentioned nerve disorders the presence of axonal polyribosomes may indicate a compensatory mechanism for proteins otherwise supplied by axonal transport, and this glial support may provide a “survival signal” that prevents axons from degeneration. On the other hand, when this glial support is compromised, e.g., in a variety of myelin diseases in PNS and CNS [10], [47], [48], axons may experience “ribosomal starvation” and ultimately degenerate. To which extent ribosomal transfer from Schwann cells to axons plays a functional role in the pathogenesis of ALS remains to be determined, but the reported finding that selective reduction of an ubiquitously expressed dismutase active SOD1 in Schwann cells in mice accelerates disease progression [49], is in line with an axon-supporting role for Schwann cells during ALS disease progression. The importance of intact myelinating glia for the survival of ALS-diseased axons was also recently demonstrated by Lee and co-workers [50]. These authors demonstrated that the lactate transporter, monocarboxylate transporter 1 (MCT1), which is expressed in oligodendroglia, is involved in lactate transport from glia to axons, and in ALS, glial MCT1 levels were found reduced, leading to reduced lactate transport to axons, which may contribute to the pathogenesis of ALS. Together, these studies emphasize the role of glial cells in maintaining axonal functioning in supplying metabolites and protein synthetic machinery to healthy and disease-compromised axons.

### Conclusions

The early appearance of axonal ribosomes, at least as observed in ALS SOD1^G93A^ mice, makes it a morphological change that may be observed before other (behavioral) markers are evident. Whether the presence of axonal ribosomes may be used as an early diagnostic marker for diverse human peripheral neuropathies is an interesting possibility that is presently under investigation.

## Supporting Information

Figure S1 **Representative images of those used for the quantification of axonal ribosomes in phrenic nerves (see** **Figure 2** **).** In A, control non-transgenic and in B, SOD1 wt, no ribosomes are detected, whereas in C, in a SOD1^G93A^ nerve, ribosomes are abundantly present. D, SOD1^G93A^, ribosomes are present in a vesicle with multiple membranes (RV, ribosome containing vesicle). M, myelin, Mt, microtubules. Bar A–D, 100 nm.
(TIF)

Figure S2 **Montage of the Z-stack of** **Figure 3** **.** Symptomatic SOD1^G93A^ mice, sciatic nerve. Nonradioactive in situ hybridization for rRNA (green) yields a strong signals in 2 myelinated axons (arrows), delineated by Nile red fluorescence of myelin (red). Optical section thickness 0.39 µm. The image shows the presence of ribosome in situ hybridization of signal through the entire Z-stack (about 10 µm) in the axon indicated by the right arrow.
(TIF)

Figure S3 **Symptomatic Sod1^G93A^ mice, sciatic nerve.** Nonradioactive in situ hybridization for rRNA (green) yields a strong signal in several myelinated axons (arrows), delineated by Nile red fluorescence of myelin (red). Bar 10, µm.
(TIF)

Figure S4 **Symptomatic Sod1^G93A^ mice, sciatic nerve.** Nonradioactive in situ hybridization for rRNA (green), myelin is stained by Nile red. Within the axonal space, the rRNA signal co-localizes with the myelin signal, suggesting that ribosomes are here confined within myelin membranes. Bar, 10 µm.
(TIF)

Figure S5 **Presymptomatic Sod1^G93A^ mice, sciatic nerve.** Mouse monoclonal (Y10B) anti-ribosomal RNA immunofluorescence (red) is present in several myelinated axons outlined by Nile red staining (arrows). Bar, 5 µm.
(TIF)
