# Supplementary figures and images for: Increased Axonal Ribosome Numbers Is an Early Event in the Pathogenesis of Amyotrophic Lateral Sclerosis
Source: PLoS One. 2014 Jan 30;9(1):e87255. doi: 10.1371/journal.pone.0087255 (PMC3907527; doi:10.1371/journal.pone.0087255)

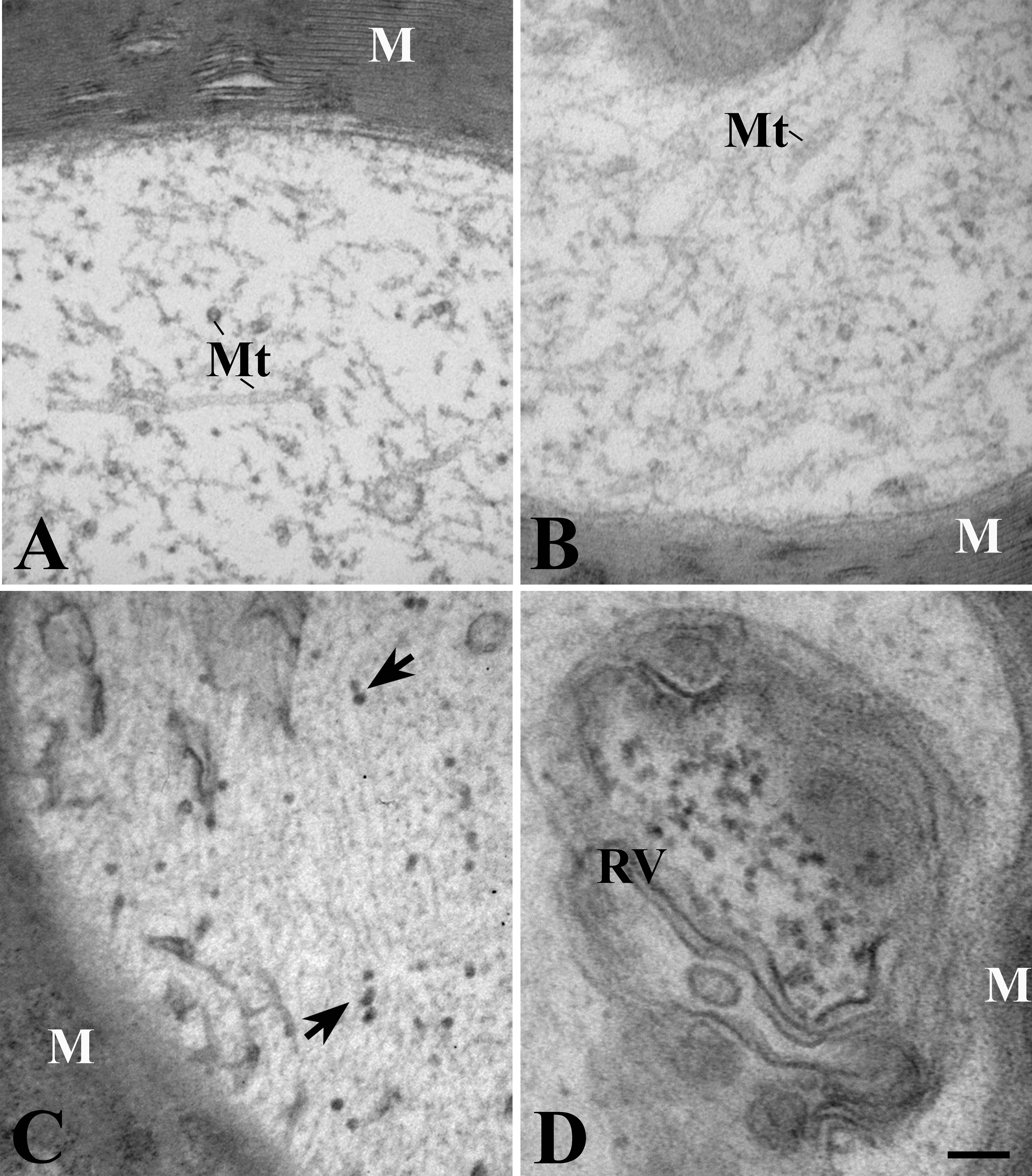

Supplement: Figure S1 — Representative images of those used for the quantification of axonal ribosomes in phrenic nerves (see Figure 2 ). In A, control non-transgenic and in B, SOD1 wt, no ribosomes are detected, whereas in C, in a SOD1G93A nerve, ribosomes are abundantly present. D, SOD1G93A, ribosomes are present in a vesicle with multiple membranes (RV, ribosome containing vesicle). M, myelin, Mt, microtubules. Bar A–D, 100 nm. (TIF) [file pone.0087255.s001.tif]

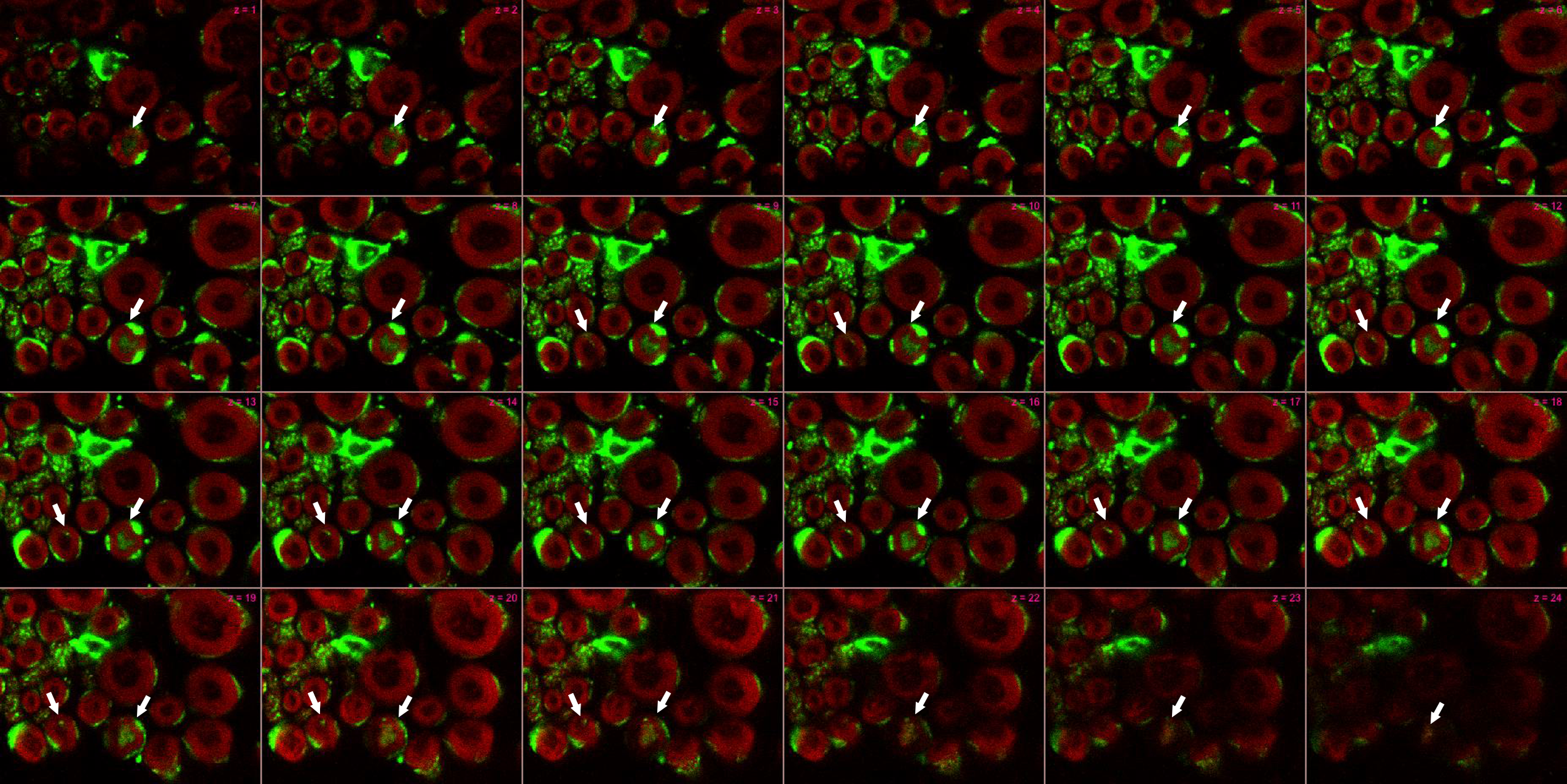

Supplement: Figure S2 — Montage of the Z-stack of Figure 3 . Symptomatic SOD1G93A mice, sciatic nerve. Nonradioactive in situ hybridization for rRNA (green) yields a strong signals in 2 myelinated axons (arrows), delineated by Nile red fluorescence of myelin (red). Optical section thickness 0.39 µm. The image shows the presence of ribosome in situ hybridization of signal through the entire Z-stack (about 10 µm) in the axon indicated by the right arrow. (TIF) [file pone.0087255.s002.tif]

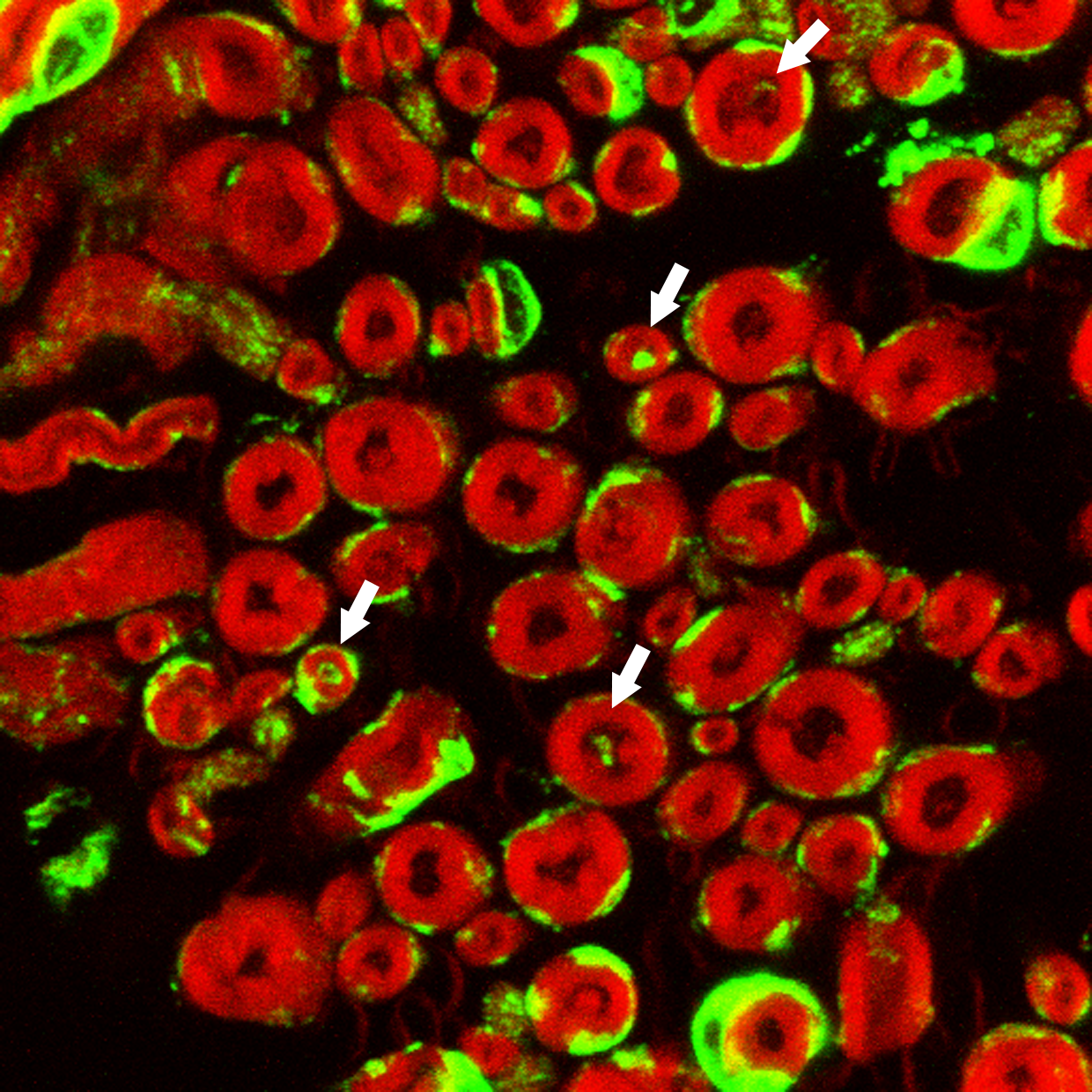

Supplement: Figure S3 — Symptomatic Sod1G93A mice, sciatic nerve. Nonradioactive in situ hybridization for rRNA (green) yields a strong signal in several myelinated axons (arrows), delineated by Nile red fluorescence of myelin (red). Bar 10, µm. (TIF) [file pone.0087255.s003.tif]

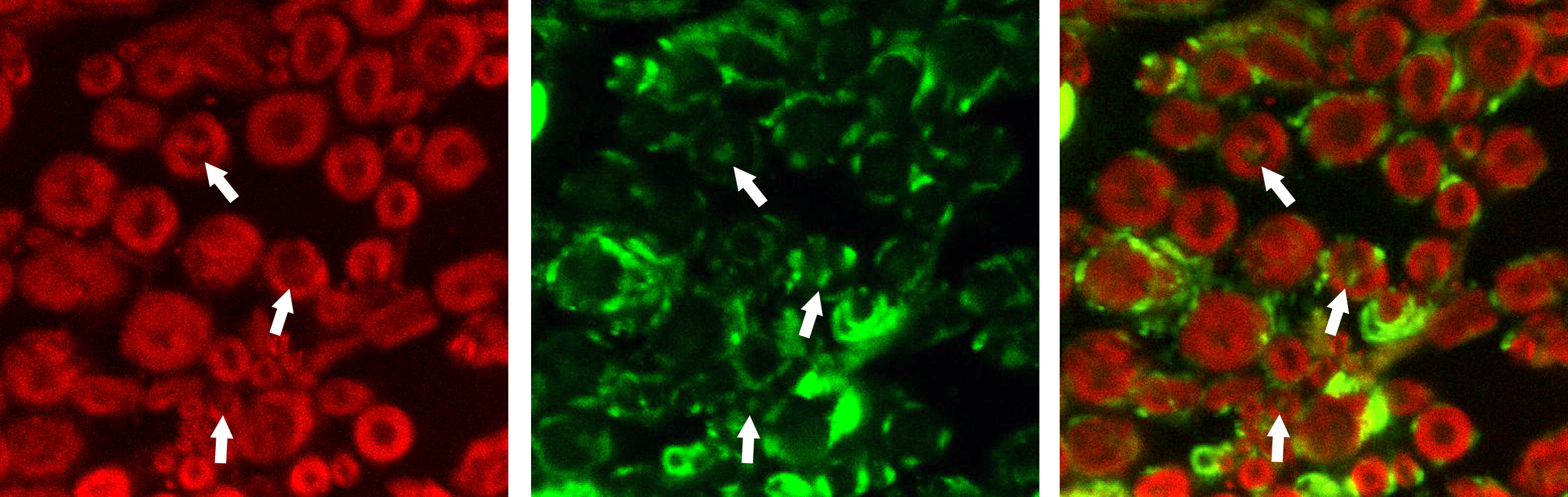

Supplement: Figure S4 — Symptomatic Sod1G93A mice, sciatic nerve. Nonradioactive in situ hybridization for rRNA (green), myelin is stained by Nile red. Within the axonal space, the rRNA signal co-localizes with the myelin signal, suggesting that ribosomes are here confined within myelin membranes. Bar, 10 µm. (TIF) [file pone.0087255.s004.tif]

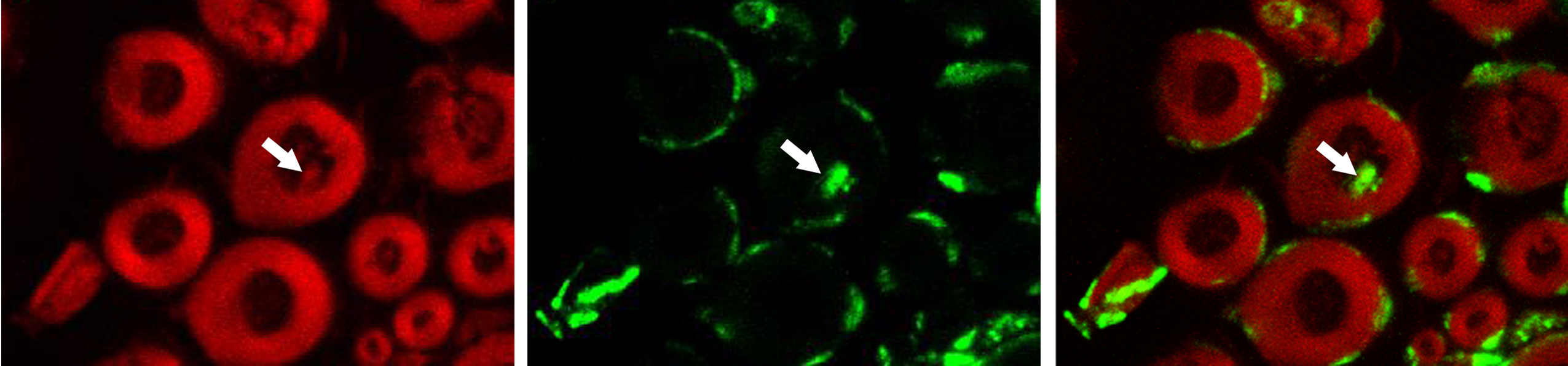

Supplement: Figure S5 — Presymptomatic Sod1G93A mice, sciatic nerve. Mouse monoclonal (Y10B) anti-ribosomal RNA immunofluorescence (red) is present in several myelinated axons outlined by Nile red staining (arrows). Bar, 5 µm. (TIF) [file pone.0087255.s005.tif]
